# Supplementary material for: Chromothripsis during telomere crisis is independent of NHEJ, and consistent with a replicative origin
Source: Genome Res. 2019 May;29(5):737–49. doi: 10.1101/gr.240705.118 (PMC6499312; doi:10.1101/gr.240705.118)
Supplement: Supplemental Material [file supp_gr.240705.118_Supplemental_file_1.zip › contigs/annotated_contigs/DB45/contig.2.DB45_length_725_mean_cov_8.19862068966.docx]

**DB45_length_725_mean_cov_8.19862068966**

```
TTCTAGTAATTTTATAGTTTCAATTCTTACATGTCAGTCTTTCATCAATTTTGAGTTAATAATTCTTTTAGCCATTTCGAGTAAGATAA
 >chr13:65808580-65808897 + E=6e-179
GAGCCTAATATTATTTTTCTGCATGCTGACATCATTTTCCCCAGCACCATTTATTAAAGAGACTGTCCTTTCCACAATGTGTGTATTTG

ATGCCTTTGTCAAAAATAAGTTTTCTGTAAGTACATGGTGTAATTTCTGGATTCTCAATTCTATTCCATTGTGCTATGTGTCTTTTTTT

ATGCCATTACCGTGCTGTTTTGGTTACTATAACCCTGTAGTACA|TTTTGA|GAACTGTCTATTCAGGTCCTTAGCCCACTTTTTGATG
                                             >chr13:65810017-65810431 + E=3e-237
GGATTATTTGGATTTTTCTTGCTGATTTGTTTTGAGTTTCTTGTAGATTCTGGATATTAGTCCTTTGTTGGATGCATAGTTTGCAAAAA

TGTTCTCCTACTCTGTGGGTTGTCTGTTGACTCTGCTCATTATTTCTTTTGCTGTGCAGAAGCTTTTTAGTTTAATTAAGTCCCTTTGT

TTTTGTTGCATTTGCTTTGGGTTCTTGGTCATGAACTCTTTGTGTCTTTAACTTTTCCTCAATGTTTTACAGTTTTCAGCATAGAGAAC

TTTCCACTTCCTTCATTAAATTTATGCCTATGTATTTTATTTTTTGTAGATTTTGTAAATGGCATTTTTTTTCTTTTTCAGGTAGTTTA

CTACTAGTGTATTAA
```
